# Combining biophysical parameters, spectral indices and multivariate hyperspectral models for estimating yield and water productivity of spring wheat across different agronomic practices

**DOI:** 10.1371/journal.pone.0212294

**Published:** 2019-03-06

**Authors:** Salah El-Hendawy, Nasser Al-Suhaibani, Salah Elsayed, Yahya Refay, Majed Alotaibi, Yaser Hassan Dewir, Wael Hassan, Urs Schmidhalter

**Affiliations:** 1 Department of Plant Production, College of Food and Agriculture Sciences, King Saud University, Riyadh, Saudi Arabia; 2 Department of Agronomy, Faculty of Agriculture, Suez Canal University, Ismailia, Egypt; 3 Evaluation of Natural Resources Department, Environmental Studies and Research Institute, Sadat City University, Menoufia, Egypt; 4 Department of Horticulture, Faculty of Agriculture, Kafrelsheikh University, Kafr El Sheikh, Egypt; 5 Department of Biology, College of Science and Humanities at Quwayiah, Shaqra University, Riyadh, Saudi Arabia; 6 Department of Agricultural Botany, Faculty of Agriculture, Suez Canal University, Ismailia, Egypt; 7 Chair of Plant Nutrition, Department of Plant Sciences, Technical University of Munich, Freising-Weihenstephan, Freising, Germany; Potsdam Institute for Climate Impact Research, GERMANY

## Abstract

Manipulating plant densities under different irrigation rates can have a significant impact on grain yield and water use efficiency by exerting positive or negative effects on ET. Whereas traditional spectral reflectance indices (SRIs) have been used to assess biophysical parameters and yield, the potential of multivariate models has little been investigated to estimate these parameters under multiple agronomic practices. Therefore, both simple indices and multivariate models (partial least square regression (PLSR) and support vector machines (SVR)) obtained from hyperspectral reflectance data were compared for their applicability for assessing the biophysical parameters in a field experiment involving different combinations of three irrigation rates (1.00, 0.75, and 0.50 ET) and five plant densities (D_1_: 150, D_2_: 250, D_3_: 350, D_4_: 450, and D_5_: 550 seeds m^-2^) in order to improve productivity and water use efficiency of wheat. Results show that the highest values for green leaf area, aboveground biomass, and grain yield were obtained from the combination of D_3_ or D_4_ with 1.00 ET, while the combination of 0.75 ET and D_3_ was the best treatment for achieving the highest values for water use efficiency. Wheat yield response factor (ky) was acceptable when the 0.75 ET was combined with D_2_, D_3_, or D_4_ or when the 0.50 ET was combined with D_2_ or D_3_, as the ky values of these combinations were less than or around one. The production function indicated that about 75% grain yield variation could be attributed to the variation in seasonal ET. Results also show that the performance of the SRIs fluctuated when regressions were analyzed for each irrigation rate or plant density specifically, or when the data of all irrigation rates or plant densities were combined. Most of the SRIs failed to assess biophysical parameters under specific irrigation rates and some specific plant densities, but performance improved substantially for combined data of irrigation rates and some specific plant densities. PLSR and SVR produced more accurate estimations of biophysical parameters than SRIs under specific irrigation rates and plant densities. In conclusion, hyperspectral data are useful for predicting and monitoring yield and water productivity of spring wheat across multiple agronomic practices.

## Introduction

Overexploitation and uncontrolled pumping of groundwater in several arid regions has resulted in a persistent decline in groundwater levels, a decrease in cropping area, and the amount of water allocated to each crop, and ultimately making sustainable agriculture difficult in these regions [**1,2**]. Saudi Arabia is a typical example of an arid region where the agricultural sector depends mainly on groundwater for irrigation and accounts for more than 80% of the irrigation water use [**2**]. Falling groundwater levels during the last two decades are now causing serious concerns in the sustainability of the agricultural sector in this country. Therefore, it is now more pressing than ever to develop water-saving strategies that will improve the marginal benefit produced per unit of water applied rather than those for enhancing crop production per unit area.

In areas under limited water supplies, irrigation water use efficiency (WUE), and grain yield (GY) can be improved by using different strategies, including adopting site-specific agronomic practices, developing drought-tolerance genotypes, and/or improving land husbandry practices. When assessing the advantages of these three strategies, the first one would be the most economically feasible, immediate, and effective measure, while the other two strategies may require greater efforts over several years to get the desired results. Several studies have reported the vital role of drought-tolerant genotypes in dynamically improving irrigation water use; however, this can only be achieved when combined with appropriate agronomic practices [**3–6**]. In this context, we propose that the implementation of appropriate agronomic practices could be an essential and economically feasible approach for improving irrigation water use and confronting the adverse effects of water deficits on crop production in drought-prone areas.

Soil evaporation (E) and canopy transpiration (T), otherwise known as evapotranspiration (ET), are critical components for revealing irrigation water consumption under field conditions. For wheat crops, E accounts for up to 40% of the total crop water use, especially during early growth stages where crop canopy coverage of the soil is small [**7–9**]. With increasing canopy size, T becomes the primary player in water loss while E becomes less important [**10**]. Because E may indirectly benefit crop growth and is directly associated with green leaf area, attempts to reduce E will become an effective method for improving GY and WUE under limited water supplies. Numerous studies have suggested different agronomic practices for reducing E [**11–13**]. Among these practices, adjusting plant density is considered one of the most important measures that could play an important regulatory role in reducing E and hence improving GY and WUE [**11**]. Plant density plays an important role in modifying canopy size that covers the soil, and this feature has the ability to modify the response of plants to different soil moisture contents. Areas with limited water supplies dictate the necessity for lower plant densities than those areas under well-watered conditions. In contrast, some studies have suggested that an increase in plant density could reduce E in areas with a water deficit because plants become shorter and have a decreased leaf area index (LAI) [**13**]. However, reduced plant densities under excessive irrigation often leads to a greater amount of dry matter per plant, and this does not maximize WUE [**11–13**]. Therefore, finding the best combinations of plant density and soil water availability may help to achieve maximum WUE and GY simultaneously by exerting positive or negative effects on ET at different stages of crop growth.

Deficit irrigation is another agronomic practice suggested for improving WUE under limited water supplies. It has been reported that the WUE of most cereal crops can be increased significantly by about 10–42% using deficit irrigation treatments when compared with full irrigation treatments [**12,14–16**]. However, it is difficult to apply this agronomic practice to C_3_ plants without an accompanying reduction in GY because their production is linearly correlated with crop ET. In wheat crop, irregular tiller development and a reduction in the different yield components are the major GY-reducing factors under deficit irrigation conditions. Interestingly, wheat crop have the ability to compensate for a reduction in one yield component by improving the other components, and as a result, such compensation can maximize on the yield potential under deficit irrigation conditions [**6,17**]. Because plant density can play an important synergistic role between different yield components, manipulating plant densities under different irrigation rates can have a significant impact on GY and WUE by integrating different yield components.

Several important agronomic parameters, such as green leaf area (GLA), aboveground dry matter accumulation (TDM), final GY, and WUE, could be used to define the best combinations of irrigation rate and plant density. These parameters can help us to understand the balance between GY and WUE under different water deficits. The GLA and TDM are good indicators for investigating the nature of vegetation growth and canopy architecture, the amount of incoming radiation absorbed by the canopy, and the vertical distribution of radiation through the canopy. They are also good predictors for estimating GY and ET [**18–22**]. Therefore, a rapid and non-destructive assessment of these parameters is of practical importance for finding the most successful combinations of plant density and water irrigation rate required for enhancing GY and WUE of wheat growing in water-stressed regions.

With the rapid development of proximal remote sensing techniques and multivariate data analysis, it is possible to conduct a simultaneous indirect assessment of multiple agronomic parameters in a rapid, efficient, and non-destructive manner [**23–27**]. To achieve this, it is important to determine the relationship between different agronomic parameters and their hyperspectral reflectance properties. Hyperspectral reflectance can produce hundreds of contiguous narrow wavebands, which allow a detailed study of various agronomic parameters under different agronomic practices and conditions. To minimize the effects of soil background and solar angle, several empirical SRIs and multivariate integration methods have been developed and used to assess agronomic parameters.

Previous studies mostly focused on estimating plant growth variables using SRIs under optimal or stress conditions for individual agronomic practices. Few studies have been conducted on the influence of multiple agronomic practices on canopy spectral reflectance or have defined the sensitive bands or SRIs for estimating measured parameters under such practices. One such study by Li-hong [**28**] reported that an increase in water irrigation rate, plant density, and nitrogen application of rice crops resulted in a decrease in canopy reflectance in the visible region (VIS) and an increase in the near-infrared region (NIR) of the spectrum. This was consistent with the findings of another study that measured canopy spectral reflectance of different winter wheat cultivars grown at varying plant densities [**29**]. Here, the NIR bands (780–1100 nm) were found to be better at differentiating between planting densities of different cultivars than VIS bands (460–730 nm) [**29**]. Wang et al. [**30**] also reported that some narrow-band SRIs were successfully used to estimate LAI, fresh weight of biomass, plant height, and chlorophyll contents for different spring wheat cultivars under different planting densities. Furthermore, Li [**31**] found that plant density and irrigation frequency had direct and significant effects on canopy spectral reflectance of winter wheat in the range of 400–900 nm, which included the VIS/NIR wavelength bands.

To overcome the strong multi-collinear and noisy variables in spectral band data and to efficiently estimate the measured parameters, multivariate integration methods such as PLSR and support vector machines (SVM) have been suggested to fulfil this goal [**23,32,33**]. Both methods deal with a large number of spectral bands or SRIs as a single index in order to improve the prediction of agronomic parameters. Therefore, with both methods, the measured parameters can be simultaneously assessed through a wide range of wavelengths from the VIS, NIR, and SWIR of the spectrum regions. PLSR is able to reduce the large number of measured collinear spectral factors to a few non-correlated latent factors, to prevent over-fitting or under-fitting the data, and to avoid redundant information [**34**]. The SVM can be used as an alternative non-linear regression method for transforming data using a kernel function in a new high-dimensional space. A predictive model is then built using a subset of representative instances called support vectors [**33**]. Thus far, there are few reports comparing the efficiency of SRIs and multivariate methods in estimating agronomic parameters measured under different combinations of multiple agronomic practices.

The objectives of this study were the following: (1) to determine the best combinations of water irrigation rate and plant density for maximizing GY and WUE of spring wheat growing under water-stressed conditions, and (2) to compare the performance of various SRIs with that of multivariate methods (PLSR and SVM) for estimating biophysical parameters (GLA, TDW, GY, and WUE) measured under different combinations of multiple agronomic practices. We aim to assess the best combinations of irrigation rate and plant density that can be used as effective and economically sustainable agronomic practices in managing spring wheat crops under limited water irrigation supplies. Rapid and non-destructive methods are necessary for detecting these combinations through estimations of biophysical parameters.

## Materials and methods

### Experimental site

The experiment was carried out during the 2016–2017 and 2017–2018 growing seasons at the Research Station of the College of Food and Agriculture Sciences, King Saud University, Saudi Arabia (24°25′N, 46°34′E; elevation 400 m). Average monthly climatic conditions at the experimental site during the whole of wheat growth are given in **Table 1**. The soil type in the experimental field is sandy loam in texture (75.1% sand, 16.1% silt, and 8.8% clay). The primary soil hydraulic characteristics had a field capacity (FC) of 0.151 m^3^ m^-3^, permanent wilting point (PWP) of 0.054 m^3^ m^-3^, and bulk density of 1.49 g cm^-3^ at a soil depth of 0.9 m. The specific suction pressure of -0.03 and -1.5 MPa was used to measure the soil water content at FC and PWP, respectively [**35**].

**Table 1:** Average monthly climatic conditions at the experimental site during the entier period of wheat growth (averaged over two seasons).

Months	Temperature (°C)			Average relative humidity (%)	Average rainfall (mm)
	Maximum	Minimum	Average		
**December**	22.2	10.6	16.4	47.0	12.0
**January**	20.2	9.0	14.6	51.0	11.9
**February**	23.4	11.2	17.3	41.0	8.0
**March**	27.7	15.2	21.5	36.0	21.0
**April**	33.4	20.4	26.9	34.0	23.8

### Treatments, experimental design, and cultural practices

The experimental treatments included three different water irrigation rates (1.00, 0.75, and 0.50 of the estimated crop evapotranspiration; ETc) and five different plant densities (150, 250, 350, 450, and 550 seeds m^-2^). The amount of irrigation water for the full irrigation treatment (1.00 ETc) was estimated by multiplying the daily reference evapotranspiration (ETo) by the crop coefficient (Kc) of spring wheat. Daily meteorological data such as net solar radiation (MJ m^2^ day^-1^), air temperature (°C), wind speed (m s^-1^) and relative humidity (%) at a 2 m height, saturation and actual vapor pressure (kPa), soil heat flux density (MJ m^2^ day^-1^), psychrometric constant (kPa °C^-1^), and slope of the saturation vapor pressure curve (kPa °C^-1^) were collected from weather stations located 200 m from the experimental site. These data were used for estimating the ETo using the FAO PenmanMonteith equation given by Allen et al. [**36**]. The values of Kc for spring wheat as recommended by FAO-56 [**36**] were adjusted using the actual values of relative humidity and wind speed in the study area. The cumulative irrigation estimated for the full irrigation treatment (1.00 ETc) was 558.0 and 555.0 mm ha^-1^ during the first and second growing seasons, respectively. The amount of irrigation water was reduced to 25 and 50% for the 0.75 and 0.50 ETc water deficit treatments, respectively. The amount of water for each irrigation treatment was applied eight times; once at seedling, tillering, stem elongation, booting, heading, anthesis, grain milk, and grain dough stages. To ensure full germination, about 38 and 45 mm ha^-1^ of water was applied at sowing to all treatments in the first and second seasons, respectively, to ensure full germination.

Irrigation was applied using a low-pressure water transportation surface irrigation system. The main water pipe of the irrigation system was equipped with a flow meter connected at the location where the main line encountered the main water source, distributed to the sub-main hoses at each subplot, and equipped with a manual control valve in order to monitor and control the amount of water delivered for each irrigation rate.

Two levels below (150 and 250 seeds m^-2^) and two levels above (450 and 550 seeds m^-2^) the standard recommended plant density (350 seeds m^-2^) for spring wheat were applied for each water irrigation rate. When the plants were between the one- and two-leaf stages, plants along 0.50 m sections of two rows in each subplot were counted in order to confirm the final number of plants per square meter for each plant density.

Field experiments were performed using a randomized complete block design with split-plot arrangements and three replications. Water irrigation rate was randomly assigned to the main plots within each replicate and plant density was randomly assigned to the split plots within each main plot. Each subplot was 6.0 m long and eight rows wide with an inter-row spacing of 0.15 m (7.2 m^2^ in total area). A 3.0 m wide isolation belt was placed between each adjacent main plot to prevent water leakage. The seeds of the spring wheat variety Sakha 94 were sown by hand on December 1^st^ in both seasons. Nitrogen, as ammonium nitrate (33.5% N); phosphorus, as calcium superphosphate (15.5% P_2_O_5_); and potassium, as potassium chloride (60% K_2_O) were applied at rates of 180, 90, and 60 kg ha^-1^, respectively. The entire amount of phosphorus was applied prior to seeding, whereas the entire dose of potassium was applied at booting stage. The nitrogen fertilizer was applied in three doses: at seeding (30%), stem elongation (40%), and booting stages (30%).

### Measurements

#### Canopy hyperspectral reflectance measurements

**Fig 1:**
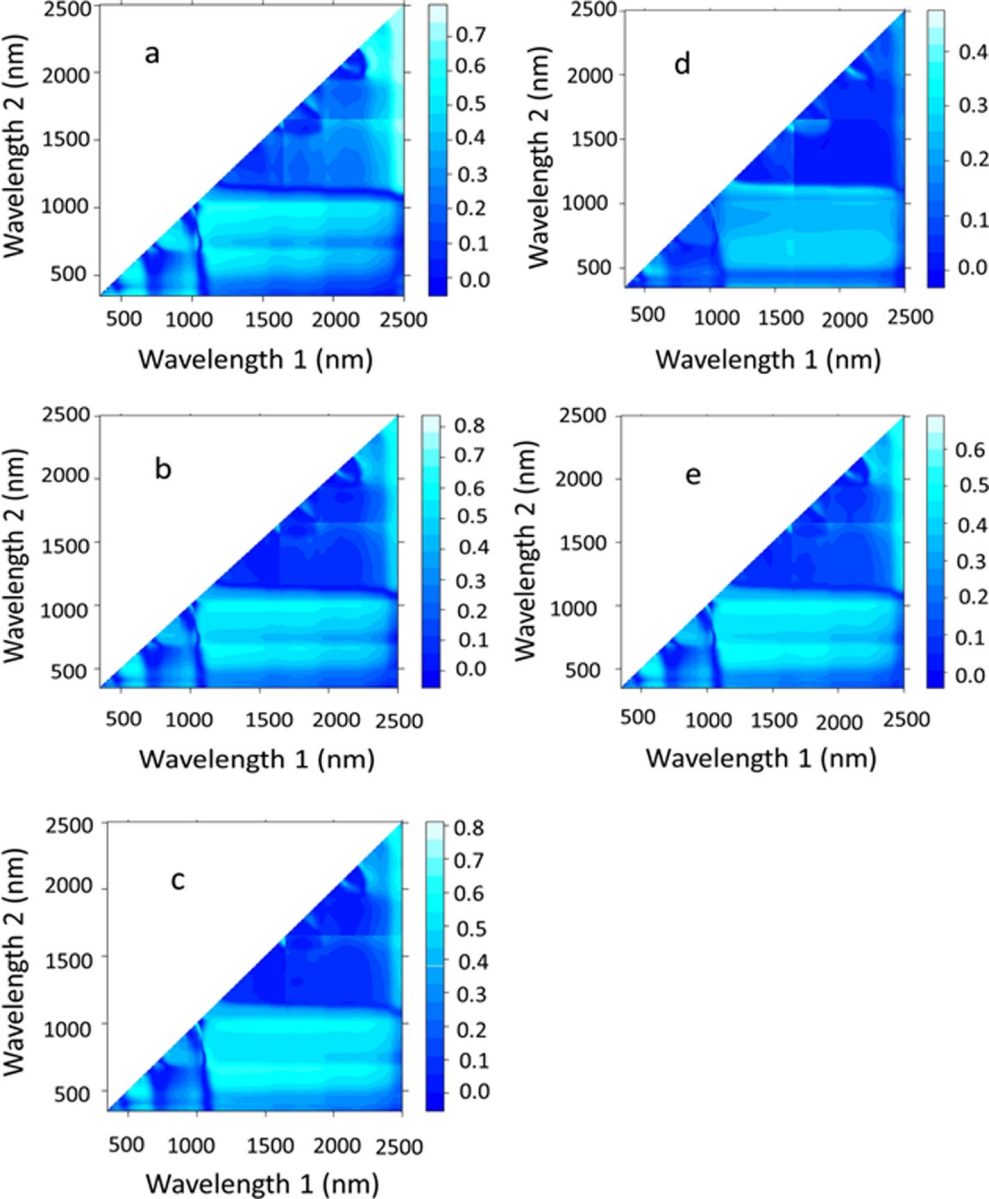
Contour maps of coefficients of determination (R²) for all dual wavelength combinations in the spectral range of 350–2500 nm as normalized difference spectral indices. The pooled data of replications, irrigation rates, plant densities, and season for each agronomic parameter i.e., green leaf area (a), aboveground dry weight (b), grain yield (c), and water use efficiency (d), and for all agronomic parameters combined were used.

Canopy reflectance spectra were measured at anthesis growth stage under sunny and windless conditions between the hours of 10.00 and 15.00 Saudi Arabia Standard (UTC+2). Within this period, the weather in the study area remained stable from anthesis to maturity stage. The solar radiation reflected from the wheat canopy was captured by a portable Field Spec spectroradiometer (Analytical Spectral Devices Inc., Boulder, CO, USA) over a 350–2500 nm spectral region. The radiation was captured at 0.8 m above the canopy with a nadir field of view (FOV) of 25° and a spectral sampling interval of 1.4 and 2.2 nm for the 350–1000 and 1000–2500 nm region, respectively. However, the entire spectral range (350–2500 nm) was calculated automatically to resample to 1.0-nm continuous bands. The 0.80 m vertical height coupled with 25° FOV covers a ~0.2 m^2^ sensing area. A 40 cm × 40 cm calibration white reference panel covered with a mixture of barium sulfate (BaSO_4_) and white paint was used to calibrate the spectroradiometer and to generate reflected light percentages. These calibrations were performed before and after the canopy spectral reflectance measurement for each subplot. The spectral data of each subplot were collected by averaging six sequential readings taken at different points on the three central rows within each subplot by excluding the first meter of each row to eliminate any border effects. Each spectral measurement was calculated automatically by averaging 10 scans at an interval of one second. Finally, the spectral data were exported to View Spec Pro (ASD) software and averaged for each subplot. SRIs were calculated based on published SRIs or newly developed SRIs extracted from contour maps (**Table 2**). The contour maps of SRI readings show the coefficients of determination (R^2^) from combinations of two individual wavelengths in the spectral range of 350–2500 nm as normalized difference indices (**Fig 1**).

**Table 2:** Full name, abbreviation (Abb.), and formula of different spectral reflectance indices (SRIs) developed in this study and from the literature.

**Full name and Abb. of SRIs**	**Formula**	**Reference**
Normalized difference Index (NDI_548,522_)	(R_548_ − R_522_) / (R_548_ + R_522_)	This work
Normalized difference Index (NDI_626,386_)	(R_626_ − R_386_) / (R_626_ + R_386_)	This work
Normalized difference Index (NDI_680,1650_)	(R_680_ − R_1650_) / (R_680_ + R_1650_)	This work
Normalized difference Index (NDI_840,818_)	(R_840_ − R_818_) / (R_840_ + R_818_)	This work
Normalized difference Index (NDI_1226,670_)	(R_1226_ –R_670_) / (R_1226_ + R_670_)	This work
Normalized difference Index (NDI_1382,670_)	(R_1382_ –R_670_) / (R_1382_ + R_670_)	This work
Normalized difference Index (NDI_1450,900_)	(R_1450_ –R_900_) / (R_1450_ + R_900_)	This work
Normalized difference Index (NDI_1650,920_)	(R_1650_ –R_920_) / (R_1650_ + R_920_)	This work
Normalized difference Index (NDI_2450,2100_)	(R_2450_ − R_2100_) / (R_2450_ + R_2100_)	This work
Normalized difference Index (NDI_2498,1450_)	(R_2498_ − R_1450_) / (R_2498_ + R_1450_)	This work
Normalized difference Index (NDI_2500,2250_)	(R_2500_ − R_2250_) / (R_2500_ + R_2250_)	This work
Normalized difference Index (NDI_2500,2470_)	(R_2500_ − R_2470_) / (R_2500_ + R_2470_)	This work
Moisture stress index (MSI)	R_1600_ /R_820_	[**37**]
Simple ratio water index (SRWI)	R_860_/R_1240_	[**38**]
Normalized water index -3 (NWI-3)	(R_970_ − R_880_) / (R_970_ + R_880_)	[**39**]
Normalized difference vegetation index (NDVI _900,685_)	(R_900_ − R_685_) / (R_900_ + R_685_)	[**40**]
Normalized difference moisture index (NDMI_2200,1100_)	(R_2200_ –R_1100_) / (R_2200_ + R_1100_)	[**41**]
Normalized multi-band drought index (NMDI)	R_860_ − (R_1640_ –R_2130_) / R_860_ + (R_1640_ + R_2130_)	[**42**]
Optimized soil adjusted vegetation index (OSAVI)	(R_800_ − R_670_)/(R_800_ + R_670_ + 0.16)	[**43**]
Modified triangular vegetation index (MTVI)	1.2 × [(1.2 × (R_800_ –R_550_)– 2.5 × (R_670_ –R_550_)]	[44]

The different SRIs were selected to cover all combinations of the three main spectrum regions [(visible-infrared (VIS), near-infrared (NIR), and shortwave-infrared (SWIR)] and incorporated the wavelengths of the spectrum that are sensitive to changes in leaf/tissue structure, leaf pigmentation, aboveground biomass, photosynthetic efficiency, and/or plant water status. For instance, SRIs such as moisture stress index (MSI), simple ratio water index.

(SRWI), normalized water index 3 (NWI-3), normalized difference moisture index (NDMI), NDI_(1450_900)_, NDI_(1650_920)_, NDI_(2498_1450)_, and NDI_(2500_2250)_ incorporated a reference spectral band where the water absorption coefficient is weak and a measured spectral band where the water absorption coefficient is moderate or high. SRIs such as NDI_(680_1650)_ and NDI_(1226_670)_ included wavelengths where one is sensitive to change in plant water status and the other is sensitive to change in leaf pigmentation and photosynthetic efficiency. The wavelengths in normalized difference vegetation index (NDVI), modified triangular vegetation index (MTVI), and optimized soil-adjusted vegetation index (OSAVI) were related to estimate aerial biomass of crops.

#### Growth measurements

After completing canopy spectral reflectance measurements, an area of 0.15 m^2^ (two 0.5 m consecutive rows) of wheat plants from each subplot was cut from ground level, placed in a plastic bag, and transported to the laboratory for aboveground biomass (TDW) measurements. The plant samples were cut into small pieces, put into drying bags, and dried in a forced-air oven at 70°C until they reached a constant weight. The TDW was estimated on a land-area basis using the width and length of the harvested area. An additional 20 plants were collected randomly from each subplot, and all green leaves were separated and run through an area meter (LI 3100; LI-COR Inc., Lincoln, NE, USA) to measure surface GLA.

When wheat plants reached maturity, an area of 3.0 m^2^ (four 5 m consecutive rows) were harvested from each subplot in order to measure grain yield (GY). Ears were separated from plants, air-dried, threshed, and finally GY was adjusted to approximately 14% moisture content.

#### Water use efficiency (WUE) and yield response factor (ky)

WUE was calculated by dividing GY by actual crop evapotranspiration (ET). ET was estimated using the general water balance equation:

ET=ETc+P+Cr−R−D±ΔS

where ETc is the amount of irrigation water estimated for each water irrigation rate (mm); P is the effective precipitation during the entire wheat growing season (mm), Cr is the capillary rise to the root zone (mm), which was estimated to be zero due to the deep level of groundwater; R is the surface runoff (mm), which was also taken as zero because the irrigations were performed with a low-pressure water transportation surface irrigation system and each subplot was surrounded by earth dikes; D is the deep percolation below the plant root zone (mm), which was estimated from the respective soil water content to a depth of 90 cm before irrigation and subtracted from the soil water hold at filed capacity; and ΔS is the difference between soil water content at sowing and harvesting, at a depth of 90 cm. Soil water content was measured by the gravimetric method. The values were converted to a volumetric basis by multiplying them by the soil depth of the soil samples and the bulk density of the respective layer.

The ky for each season and for each combination of plant density and deficit irrigation rate (0.75 and 0.50 ETc) was determined through the slope of the regression between the relative GY decrease (1−GYa/GYm) and the corresponding relative seasonal crop evapotranspiration (ET) deficit (1−ETa/ETm) [**45**], where GYa and GYm are the actual and maximum GY values, respectively, and ETa and ETm are the corresponding actual and maximum ET values, respectively.

### Data analysis

Data for the different agronomic parameters (GLA, TDW, GY, and WUE) were tested using analysis of variance appropriate for a randomized complete block split-plot design, with irrigation rate as the main factor and plant density as the split factor. The difference between the different mean values of these parameters were compared using Duncan’s test at the 95% probability level. The relationships between the 20 published and newly developed SRIs and measured parameters were calculated using simple regression analysis (Sigma Plot 11.0).

The Unscrambler X multivariate data analysis software version 10.2 (CAMO Software AS, Oslo) was used to calibrate and validate the models of PLSR and SVM. Both methods (PLSR and SVM) were applied to extract the information concerning the measured agronomic parameters as well as to take the strong collinearity of spectral bands into account and to increase the prediction of the measured parameters. The full VIS-SWIR spectrum regions (350–2500 nm) were utilized in both methods across two years to predict the measured parameters at each plant density, irrigation rate, and for each year. Cross validation was done through all the data of two years for each measured parameter in order to select the best calibration models based on lowest root mean square errors (RMSE) and optimal latent factor. Then the calibration model was used as an independent model for validating the data at each plant density, irrigation rate, and for each year. The reliability of the two models for the different agronomic parameters was expressed through adjusted coefficients of determination (R^2^), RMSE, and slopes of the relationships between the observed and predicted values of each agronomic parameter.

## Results

### Growth, GY, and WUE

**Fig 2:**
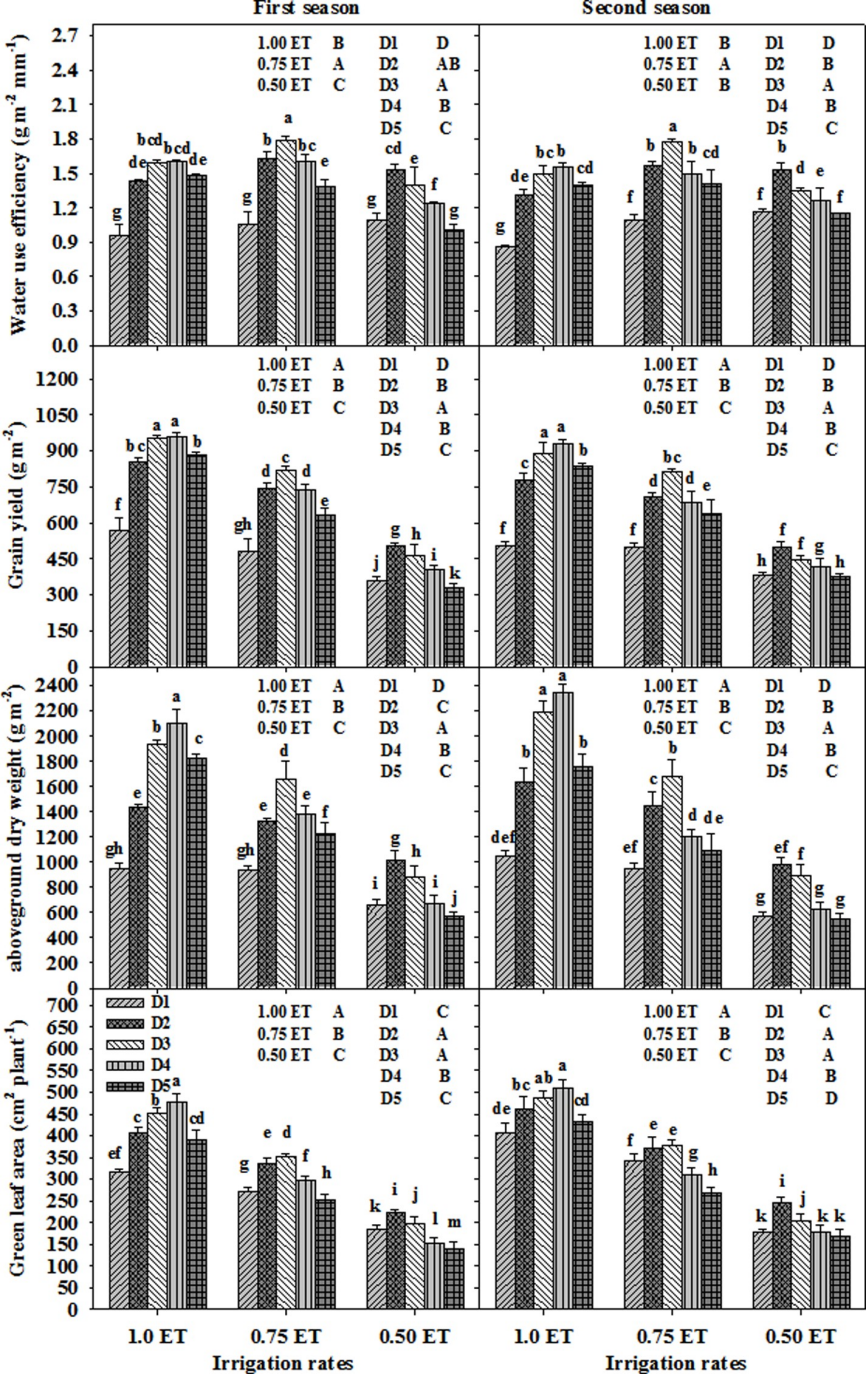
The effects of combining different irrigation rates with different plant densities on measured agronomic parameters in two growing seasons. Bars with different letters are significantly different from each another at P ≤ 0.05. Vertical bars indicate standard error (n = 3). D_1_, D_2_, D_3_, D_4_, and D_5_ indicate plant densities of 150, 250, 350, 450, and 550 seeds m^-2^, respectively.

Irrigation rates combined with different plant densities exerted a significant effect on the measured agronomic parameters, namely GLA, aboveground TDW, GY, and WUE, in both growing seasons (**Fig 2**). Generally, moderate (0.75 ET) and low (0.50 ET) irrigation rates led to significantly decreased growth parameters (GLA and TDW) and GY than full irrigation rate (1.00 ET). However, 0.75 ET displayed the highest WUE in both growing seasons, with 0.50 ET having a similar WUE to 1.00 ET in the second season **(Fig 2**).

The measured parameters were also significantly affected by plant density. The highest values for all parameters were obtained at plant densities of 350 seeds m^-2^ (D_3_) followed by densities of 250 seeds m^-2^ (D_2_) or 450 seeds m^-2^ (D_4_). The very low (150 seeds m^-2^, D_1_) and very high (550 seeds m^-2^, D_5_) plant densities exhibited the lowest values for all measured parameters (**Fig 2**).

The combined effects of irrigation rate and plant density had a significant impact on all measured parameters in both growing seasons. Generally, densities of D_3_ or D_4_, D_3_, and D_2_ were the best densities to combine with irrigation rates of 1.00, 0.75, and 0.50 ET, respectively (**Fig 2**). Interestingly, the values of TDW, GY, and WUE obtained from the combination of 0.75 ET (25% reduction in the quantity of water applied) and D_3_ were higher by 42.8, 30.7, and 46.1% in the first season and 37.8, 37.7, and 51.6% in the second season, respectively, than those achieved from the combination of full irrigation and D_1_. Importantly, the combination of 0.50 ET (50% reduction in the quantity of water applied) and D_2_ or D_3_ produced TDW and GY values similar to those obtained from the combination of D_1_ and 1.00 or 0.75 ET. The combination of 0.75 ET and D_3_ was the best treatment for achieving the highest values for WUE in both seasons, while combinations of 0.75 ET and D_2_ or D_4_ and 1.00 ET and D_3_ or D_4_ were comparable and not significantly different from one another (**Fig 2**).

#### Yield response factor (ky)

**Fig 3:**
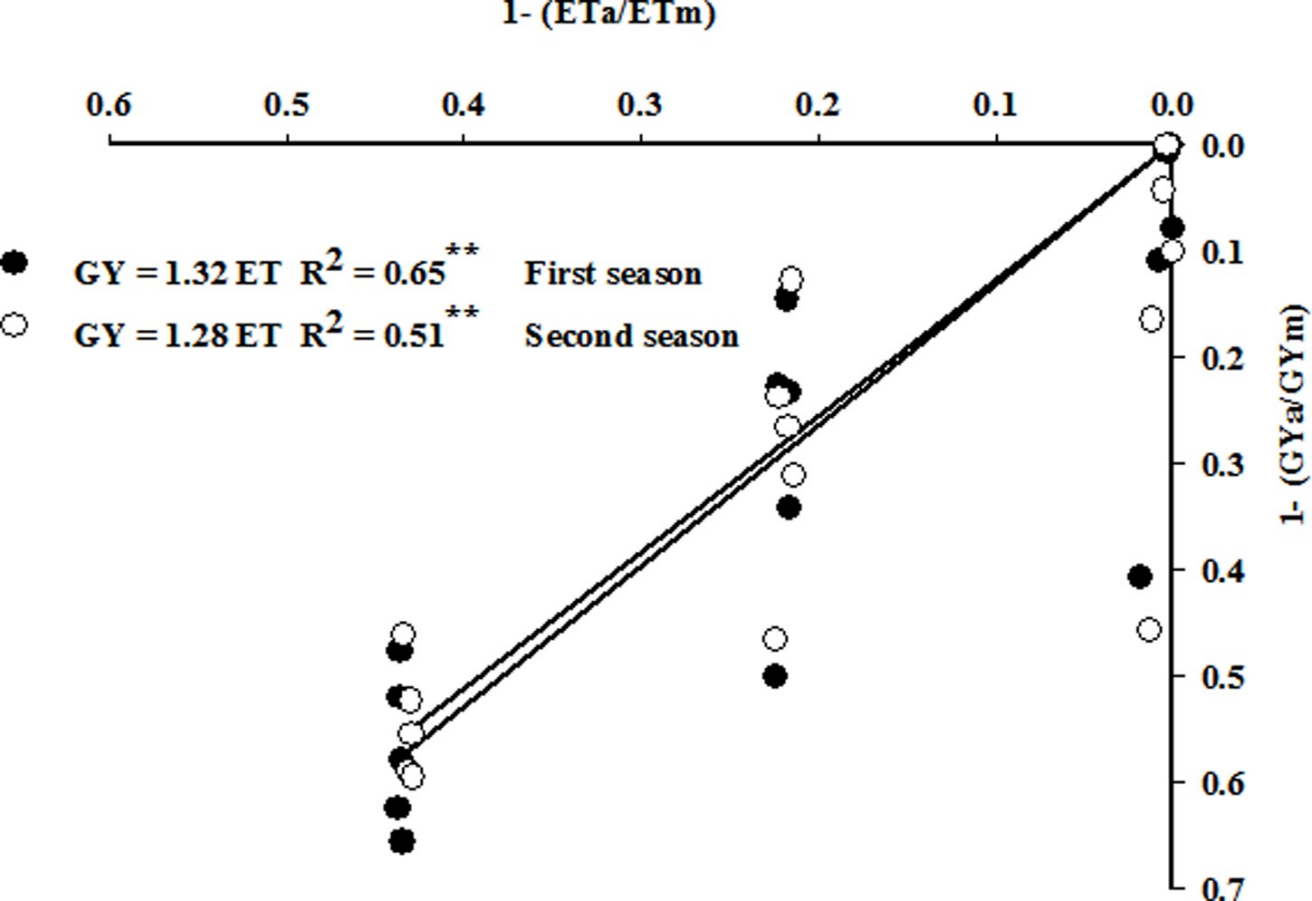
Yield response factor (ky) for two growing seasons of spring wheat under limited water supply treatments (0.75 and 0.50 ET). ** indicates significance at 0.05 P level.

Fig 3 shows the relationship between the relative GY decreases and the corresponding relative ET deficits in the two growing seasons. These relationships were linear for the pooled data of irrigation rate and plant density, with the ky values (slopes) of 1.32 and 1.28 in the first and second seasons, respectively (**Fig 3**). Moreover, **Table 3**shows the ky values for deficit irrigation treatments (0.75 or 0.50 ET) when combined with the different plant densities. The 0.75 ET produced the lowest values for ky (less than one) when it was combined with D_3_. The 0.50 ET produced comparable ky values as did the 0.75 ET, where ky values of both were slightly higher than one when both treatments were combined with D_2_. Interestingly, the ky values of 0.50 ET were significantly lower than those of 0.75 ET when they were combined with low (D_1_) and high (D_5_) plant densities, but the values of ky for these combinations were significantly higher than one (**Table 3**).

**Table 3:** Yield response factor (ky) for the combination of deficit irrigation treatments (I) (0.75 and 0.50 ET) with different plant densities (D) in two growing seasons.

I	D	First season			Second season		
		1– (ETa/ETm)	1– (GYa/GYm)	ky	1– (ETa/ETm)	1– (GYa/GYm)	ky
**0.75 ET**	**D1**	0.224	0.501	**2.23**	0.224	0.466	**2.08**
	**D2**	0.222	0.228	**1.02**	0.222	0.237	**1.07**
	**D3**	0.217	0.145	**0.67**	0.214	0.128	**0.59**
	**D4**	0.216	0.233	**1.08**	0.217	0.266	**1.13**
	**D5**	0.216	0.341	**1.58**	0.214	0.312	**1.46**
**0.50 ET**	**D1**	0.437	0.624	**1.43**	0.432	0.590	**1.37**
	**D2**	0.436	0.477	**1.10**	0.434	0.462	**1.06**
	**D3**	0.436	0.520	**1.19**	0.430	0.524	**1.22**
	**D4**	0.435	0.579	**1.33**	0.429	0.555	**1.29**
	**D5**	0.434	0.656	**1.51**	0.428	0.595	**1.39**

**D**_**1**_**, D**_**2**_**, D**_**3**_**, D**_**4**_**, and D**_**5**_ **indicate plant density of 150, 250, 350, 450, and 550 seeds m**^**-2**^**.**

#### Yield–seasonal crop ET relationship

**Fig 4:**
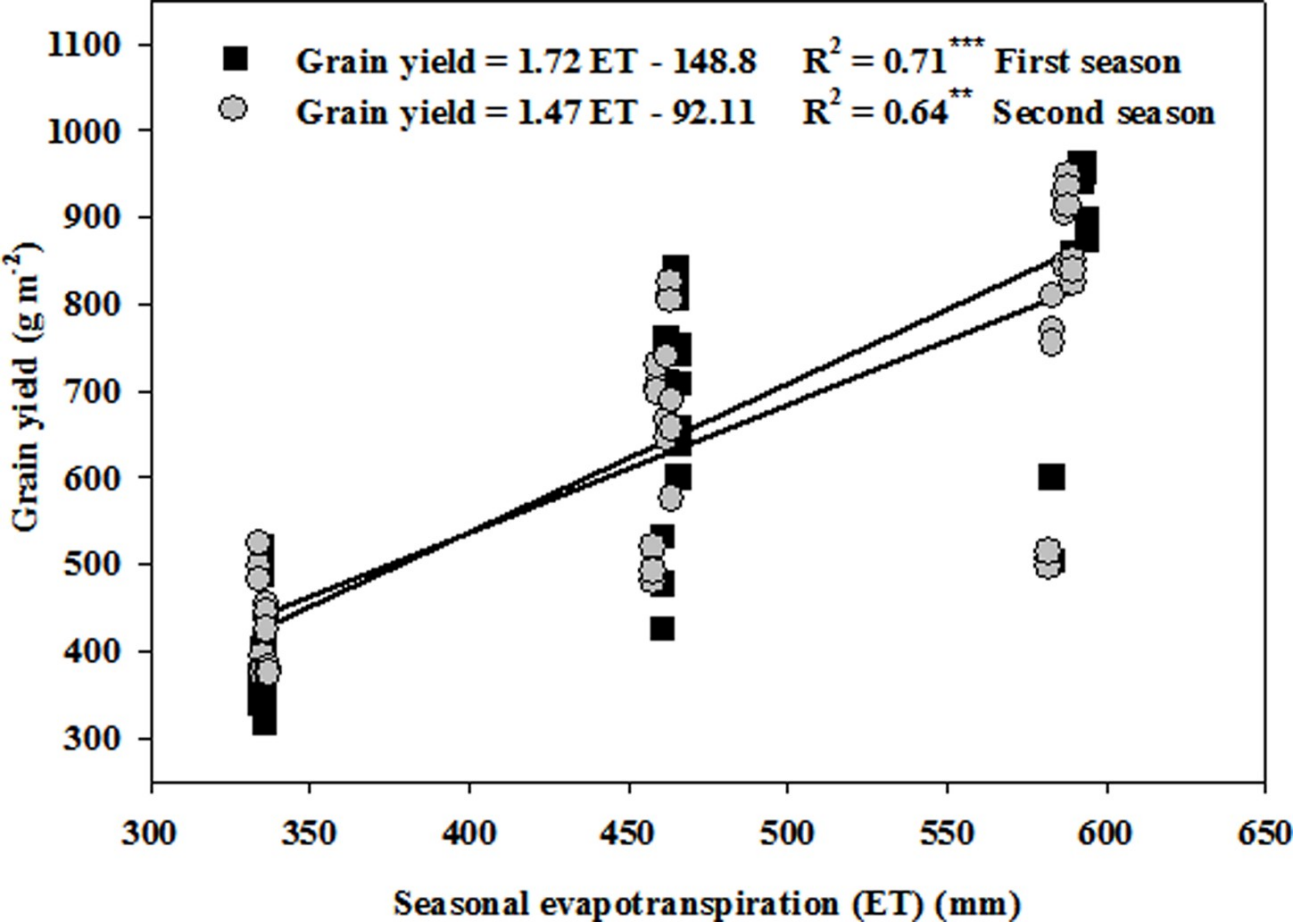
Relationship between grain yield and seasonal crop evapotranspiration of spring wheat under limited water supplies. ***, ** indicates significance at 0.001 and 0.01 P levels, respectively.

The linear model delivered the best fit for describing the relationship between GY and seasonal crop evapotranspiration. This relationship was significant for each season, with regression coefficients (R^2^) of 0.71 and 0.64 and slopes of the linear regression, which represent the increase in GY for each unit increase in ET, of 1.72 and 1.47 g m^-2^ mm^-1^ in the first and second seasons, respectively (**Fig 4**). Based on the intercepts and slopes of the regression, the basal seasonal crop ET necessary to start GY production was calculated to be 86.5 and 62.7 mm in the first and second seasons, respectively (**Fig 4**).

### Hyperspectral assessment of growth, GY, and WUE

#### Contour map analysis of the spectral reflectance data

To select the optimal SRIs for estimating the measured agronomic parameters, several contour maps were established using the pooled data of replications, irrigation rates, plant densities, and seasons for each parameter (**Fig 1A–1D**), and for all agronomic parameters combined (**Fig 1E**). These maps show the coefficients of determination (R^2^) for the relationships between all possible dual wavelength combinations of binary in the entire spectrum range (350–2500 nm) as normalized difference spectral indices and the values of measured parameters. Using the R^2^ values obtained from the contour maps, hotspot regions for the best R^2^ were located at 350–600 nm on the vertical axis with 500–700 nm on the horizontal axis; 500–1000 nm on the vertical axis with 1100–2300 nm on the horizontal axis; and 1100–2500 nm on the vertical axis with 2450–2500 nm on the horizontal axis (**Fig 1**). In general, the contour maps established for GLA, TDW, and GY (**Fig 1A, 1B and 1C**) or for all agronomic parameters combined (**Fig 1E**) showed higher R^2^ values than those established for WUE (**Fig 1D**).

#### Relationships between measured agronomic parameters and SRIs

The relationships between 20 different SRIs (12 indices developed in this study and eight indices published in the literature) and the measured agronomic parameters were analyzed under each irrigation rate (across five plant densities for two seasons, n = 10) and plant density (across three irrigation rates for two seasons, n = 6), all irrigation rates (averaged for five plant densities within each irrigation rate for two seasons, n = 6) and plant densities (averaged for three irrigation rates within each plant density for two seasons, n = 10), and all pooled data (n = 30) (**Tables 4 and 5**).

**Table 4:** The determination coefficients of the relationships between the measured agronomic parameters (green leaf area and aboveground total dry weight) and 20 developed and published spectral reflectance indices (SRIs) under each irrigation rate (n = 10) and plant density (n = 6), pooled irrigation rates (n = 6) and plant densities (n = 10), and all pooled data (n = 30).

	Irrigation rates				Plant densities (D)						Pooled data
SRIs	1.0 ET	0.75 ET	0.50 ET	All ET	D1	D2	D3	D4	D5	All D	
	Green leaf area (GLA)										
**NDI**_**(548_522)**_	**0.39**	0.01	0.02	**0.87**	0.39	**0.72**	0.41	0.23	0.01	0.09	**0.21**
**NDI**_**(626_386)**_	0.25	0.01	0.19	**0.50**	0.26	0.08	0.40	0.12	**0.58**	0.35	**0.26**
**NDI**_**(680_1650)**_	0.01	0.02	0.03	**0.55**	0.44	0.40	0.11	**0.71**	0.34	**0.86**	**0.38**
**NDI**_**(840_818)**_	0.02	0.04	**0.57**	**0.70**	0.29	0.03	0.06	**0.89**	0.39	0.02	0.10
**NDI**_**(1226_670)**_	0.02	0.00	0.18	**0.65**	0.48	0.41	0.20	**0.93**	0.45	**0.86**	**0.51**
**NDI**_**(1382_670)**_	0.01	0.02	0.04	**0.54**	0.40	0.37	0.14	**0.82**	0.32	**0.87**	**0.39**
**NDI**_**(1450_900)**_	0.12	0.06	0.26	**0.92**	**0.69**	0.43	**0.58**	0.46	**0.77**	0.19	**0.42**
**NDI**_**(1650_920)**_	0.03	0.11	0.22	**0.88**	0.47	0.02	0.34	0.45	**0.76**	0.03	**0.27**
**NDI**_**(2450_2100)**_	**0.43**	0.00	0.29	**0.96**	**0.67**	0.24	0.27	0.47	**0.65**	**0.62**	**0.41**
**NDI**_**(2498_1450)**_	**0.50**	0.00	0.20	**0.92**	0.33	0.00	0.15	0.46	**0.66**	0.06	**0.26**
**NDI**_**(2500_2250)**_	**0.55**	0.00	0.34	**0.98**	**0.75**	0.27	0.40	**0.54**	**0.69**	**0.66**	**0.48**
**NDI**_**(2500_2470)**_	**0.64**	0.02	0.34	**0.98**	0.48	0.08	0.11	**0.66**	0.41	**0.50**	**0.37**
**MSI**	0.03	0.11	0.12	**0.74**	**0.52**	0.01	0.36	0.36	**0.76**	0.01	**0.20**
**SRWI**	0.01	0.09	0.30	**0.92**	**0.52**	0.05	0.17	**0.56**	**0.62**	0.02	0.11
**NWI-3**	0.06	0.23	**0.38**	0.43	0.17	0.10	0.07	**0.55**	**0.82**	0.07	0.11
**NDVI**	0.01	0.00	0.29	**0.67**	**0.56**	0.40	0.25	**0.85**	**0.54**	**0.52**	**0.54**
**NDMI**	0.15	0.10	0.21	**0.87**	**0.51**	0.01	0.48	0.42	**0.84**	0.07	**0.30**
**NMDI**	0.01	0.11	**0.45**	**0.85**	0.40	0.07	0.27	**0.72**	**0.71**	0.04	**0.27**
**OSAVI**	0.12	0.00	0.24	**0.70**	**0.58**	0.38	0.21	**0.80**	**0.52**	**0.61**	**0.53**
**MTVI**	**0.46**	0.16	0.03	**0.53**	**0.67**	0.15	0.00	0.49	0.08	0.03	**0.17**
	**Aboveground total dry weight (TDW)**										
**NDI**_**(548_522)**_	0.18	0.10	0.00	**0.81**	0.41	**0.54**	0.39	0.21	0.001	0.26	**0.15**
**NDI**_**(626_386)**_	0.33	0.16	0.25	**0.62**	0.35	0.10	0.45	0.13	**0.71**	0.03	**0.23**
**NDI**_**(680_1650)**_	0.03	0.01	0.12	**0.66**	**0.65**	0.28	0.13	**0.68**	**0.50**	**0.82**	**0.35**
**NDI**_**(840_818)**_	0.03	0.00	**0.49**	**0.59**	0.20	0.03	0.05	**0.94**	0.30	0.03	0.07
**NDI**_**(1226_670)**_	0.04	0.00	**0.40**	**0.76**	**0.69**	0.29	0.22	**0.93**	**0.61**	0.37	**0.44**
**NDI**_**(1382_670)**_	0.03	0.00	0.14	**0.66**	**0.63**	0.25	0.16	**0.79**	0.48	**0.64**	**0.35**
**NDI**_**(1450_900)**_	0.00	0.10	0.33	**0.94**	**0.65**	0.34	**0.53**	0.49	**0.76**	0.02	**0.29**
**NDI**_**(1650_920)**_	0.01	0.14	0.25	**0.87**	0.31	0.01	0.29	**0.50**	**0.73**	0.14	**0.17**
**NDI**_**(2450_2100)**_	0.13	0.04	**0.40**	**0.99**	**0.63**	0.14	0.26	0.46	**0.64**	0.08	**0.32**
**NDI**_**(2498_1450)**_	0.11	0.11	0.25	**0.95**	0.20	0.001	0.13	0.47	**0.66**	0.05	**0.19**
**NDI**_**(2500_2250)**_	0.23	0.05	**0.41**	**0.99**	**0.67**	0.15	0.38	**0.53**	**0.69**	0.12	**0.37**
**NDI**_**(2500_2470)**_	**0.48**	0.04	**0.38**	**0.98**	0.34	0.01	0.11	**0.64**	0.46	**0.42**	**0.35**
**MSI**	0.01	0.13	0.17	**0.78**	0.33	0.01	0.30	0.41	**0.72**	0.13	**0.14**
**SRWI**	0.06	0.07	0.31	**0.85**	0.24	0.04	0.14	**0.62**	**0.54**	0.13	0.12
**NWI-3**	0.00	0.00	0.37	0.41	0.001	0.11	0.04	**0.61**	**0.67**	0.03	0.11
**NDVI**	0.02	0.00	**0.53**	**0.78**	**0.75**	0.29	0.26	**0.88**	**0.70**	0.08	**0.45**
**NDMI**	0.00	0.11	0.26	**0.89**	0.36	0.01	0.43	0.45	**0.84**	0.09	**0.19**
**NMDI**	0.02	0.11	0.37	**0.75**	0.21	0.04	0.22	**0.76**	**0.64**	0.15	**0.13**
**OSAVI**	0.12	0.00	**0.50**	**0.80**	**0.75**	0.27	0.23	**0.84**	**0.64**	0.24	**0.48**
**MTVI**	**0.38**	0.00	0.19	**0.58**	**0.65**	0.09	0.001	**0.57**	0.06	**0.40**	**0.23**

**The bold values indicate significant correlations at 0.05, 0.01 or 0.001. The full name of the abbreviations of SRI is listed in Table 2. D1, D2, D3, D4, and D5 indicate plant density of 150, 250, 350, 450, and 550 seeds m**^**-2**^**.**

**Table 5:** The determination coefficients of the relationships between the measured agronomic parameters (grain yield and water use efficiency) and 20 developed and published spectral reflectance indices (SRIs) under each irrigation rate (n = 10) and plant density (n = 6), pooled irrigation rates (n = 6) and plant densities (n = 10), and all pooled data (n = 30).

	Irrigation rates				Plant densities (D)						Pooled data
SRIs	1.0 ET	0.75 ET	0.50 ET	All ET	D1	D2	D3	D4	D5	All D	
	Grain yield (GY)										
**NDI**_**(548_522)**_	0.12	0.09	0.00	**0.73**	0.41	**0.50**	0.19	0.22	0.01	0.20	**0.15**
**NDI**_**(626_386)**_	0.15	0.08	0.19	**0.57**	0.36	0.22	0.43	0.10	**0.63**	0.04	**0.23**
**NDI**_**(680_1650)**_	0.15	0.01	0.11	**0.66**	**0.75**	0.44	0.14	**0.69**	0.40	**0.73**	**0.37**
**NDI**_**(840_818)**_	0.02	0.07	0.27	**0.47**	0.01	0.12	0.03	**0.96**	0.34	0.03	0.04
**NDI**_**(1226_670)**_	0.14	0.00	0.27	**0.76**	**0.79**	0.45	0.21	**0.99**	**0.51**	0.29	**0.44**
**NDI**_**(1382_670)**_	0.14	0.00	0.11	**0.66**	**0.73**	0.41	0.17	**0.79**	0.38	**0.52**	**0.37**
**NDI**_**(1450_900)**_	0.00	0.18	0.19	**0.89**	**0.76**	0.31	0.32	**0.56**	**0.75**	0.03	**0.26**
**NDI**_**(1650_920)**_	0.05	0.30	0.13	**0.81**	0.36	0.03	0.15	**0.54**	**0.72**	0.15	**0.13**
**NDI**_**(2450_2100)**_	0.13	0.04	0.26	**0.91**	**0.62**	0.14	0.17	**0.56**	**0.58**	0.07	**0.30**
**NDI**_**(2498_1450)**_	0.03	0.14	0.12	**0.82**	0.10	0.01	0.05	**0.55**	**0.57**	0.06	**0.15**
**NDI**_**(2500_2250)**_	0.20	0.05	0.28	**0.91**	**0.64**	0.16	0.24	**0.62**	**0.63**	0.09	**0.35**
**NDI**_**(2500_2470)**_	**0.44**	0.03	0.25	**0.90**	0.21	0.02	0.01	**0.72**	0.42	**0.47**	**0.31**
**MSI**	0.05	0.30	0.06	**0.73**	0.34	0.03	0.17	0.46	**0.73**	0.13	**0.14**
**SRWI**	0.10	0.23	0.17	**0.75**	0.24	0.11	0.06	**0.65**	**0.54**	0.13	0.08
**NWI-3**	0.03	0.05	0.22	0.27	0.001	0.22	0.001	**0.61**	**0.65**	0.03	0.06
**NDVI**	0.09	0.01	0.30	**0.77**	**0.85**	0.45	0.24	**0.93**	**0.60**	0.06	**0.45**
**NDMI**	0.06	0.21	0.13	**0.81**	0.37	0.001	0.23	**0.51**	**0.80**	0.10	**0.16**
**NMDI**	0.05	0.27	0.28	**0.68**	0.25	0.07	0.10	**0.76**	**0.65**	0.16	**0.19**
**OSAVI**	0.20	0.00	0.27	**0.76**	**0.81**	0.37	0.18	**0.89**	**0.55**	0.20	**0.45**
**MTVI**	0.18	0.01	0.07	**0.58**	0.49	0.01	0.05	**0.59**	0.04	**0.46**	**0.17**
	**Water use efficiency (WUE)**										
**NDI**_**(548_522)**_	0.12	0.10	0.00	0.23	0.02	**0.76**	0.04	0.15	0.05	0.16	0.01
**NDI**_**(626_386)**_	0.15	0.09	0.19	0.06	**0.78**	0.04	0.12	0.05	0.43	0.09	0.04
**NDI**_**(680_1650)**_	0.15	0.001	0.11	0.10	0.39	0.47	0.03	0.49	0.26	**0.72**	0.05
**NDI**_**(840_818)**_	0.02	0.05	0.28	0.06	0.05	0.09	0.06	**0.82**	0.42	0.01	0.01
**NDI**_**(1226_670)**_	0.15	0.001	0.27	0.16	0.33	0.49	0.03	**0.81**	0.36	0.39	0.06
**NDI**_**(1382_670)**_	0.14	0.001	0.11	0.10	0.34	0.47	0.04	**0.56**	0.24	**0.60**	0.04
**NDI**_**(1450_900)**_	0.00	0.19	0.19	0.27	0.19	**0.78**	0.03	**0.55**	**0.68**	0.003	0.04
**NDI**_**(1650_920)**_	0.05	0.27	0.13	0.23	0.03	0.001	0.07	0.49	**0.65**	0.07	0.01
**NDI**_**(2450_2100)**_	0.13	0.04	0.26	0.20	0.15	**0.82**	0.08	**0.56**	0.42	0.14	0.06
**NDI**_**(2498_1450)**_	0.04	0.14	0.12	0.15	0.03	**0.61**	0.23	**0.56**	0.35	0.02	0.02
**NDI**_**(2500_2250)**_	0.20	0.05	0.28	0.18	0.20	**0.82**	0.07	**0.61**	0.48	0.16	0.06
**NDI**_**(2500_2470)**_	**0.44**	0.04	0.25	0.19	0.03	**0.58**	0.28	**0.64**	0.37	**0.55**	0.09
**MSI**	0.05	0.27	0.06	0.24	0.04	0.06	0.05	0.45	**0.69**	0.07	0.01
**SRWI**	0.09	0.19	0.17	0.10	0.09	0.01	0.13	**0.55**	**0.50**	0.06	0.002
**NWI-3**	0.03	0.03	0.22	0.02	0.05	0.02	0.29	0.47	0.47	0.004	0.004
**NDVI**	0.10	0.01	0.30	0.16	0.42	0.49	0.01	**0.78**	0.44	0.13	0.06
**NDMI**	0.05	0.19	0.13	0.23	0.06	0.43	0.04	**0.51**	**0.65**	0.04	0.02
**NMDI**	0.05	0.23	0.28	0.11	0.01	0.01	0.09	**0.54**	**0.62**	0.08	0.001
**OSAVI**	0.21	0.00	0.27	0.13	0.42	**0.63**	0.001	**0.77**	0.41	0.29	0.07
**MTVI**	0.18	0.01	0.07	0.001	0.48	**0.91**	0.40	**0.55**	0.03	**0.49**	0.05

**The bold values indicate significant correlations at 0.05, 0.01 or 0.001. The full name of the abbreviations of SRI is listed in Table 2. D1, D2, D3, D4, and D5 indicate plant density of 150, 250, 350, 450, and 550 seeds m**^**-2**^**.**

In general, the results showed that under specific irrigation rates, all the SRIs examined failed to assess all agronomic parameters under 0.75 ET, and GY and WUE under 0.50 ET. There were a few SRIs (a maximum of seven out of 20 SRIs) showing a moderate relationship with GLA and TDW at 1.00 and 0.50 ET treatments (**Table 4**). For instance, the SRIs such as NDI_(548,522)_, NDI_(2450,2100)_, NDI_(2498,1450)_, NDI_(2500,2250)_, NDI_(2500,2470)_ and MTVI at 1.00 ET, and NDI_(840,818)_, NDI_(1226,670)_, NDI_(2450,2100)_, NDI_(2500,2250)_, NDI_(2500,2470)_, NDVI and OSAVI at 0.50 ET showed moderate relationships with GLA (R^2^ = 0.39–0.64) and TDW (R^2^ = 0.38–0.53), respectively (**Table 4**). Interestingly, all the SRIs examined, except for NWI-3, showed moderate to strong relationships with GLA (R^2^ = 0.50–0.98), TDW (R^2^ = 0.58–0.99), and GY (R^2^ = 0.47–0.91) when the data of all three irrigation rates were combined; however, they failed to estimate WUE (**Tables 4 and 5**).

It is notable that four out of 20 SRIs for TDW, GY, and WUE (i.e. NDI_(680,1650)_, NDI_(1382,670)_, NDI_(2500,2470),_ and MTVI) and eight out of 20 SRIs for GLA (i.e. NDI_(680,1650)_, NDI_(1226,670)_, NDI_(1382,670)_, NDI_(2450,2100)_, NDI_(2500,2250)_, NDI_(2500,2470)_, NDVI, and OSAVI) showed moderate to strong relationships with these parameters when regressions were analyzed for the combined data of all plant densities (**Tables 4 and 5**). Under specific plant density, the majority of SRIs exhibited moderate to strong (R^2^ values ranging from 0.50 to 0.99) relationships with GLA, TDW, and GY under D_4_ and D_5_ and with WUE (R^2^ values ranging from 0.51 to 0.82) under D_4_. A sufficient number of SRIs (about half) still showed moderate to strong (R^2^ values ranging from 0.51 to 0.85) relationships with GLA, TDW, and GY under D_1_ and with WUE (R^2^ values ranging from 0.58 to 0.91) under D_1_. All the SRIs failed to estimate the variations in GY and WUE under D_3_, and only one SRI (NDI_(548_522)_) that showed moderate relationships with GLA, TDW, and GY under D_2_ as well as NDI_(1450_900)_ with GLA and TDW under D_3_, and NDI_(626_386)_ with WUE under D_1_ (Tables 4 and 5). All SRIs, except three (NDI_(840,818)_, SRWI, and NWI-3), showed weak to moderate (R^2^ values ranging from 0.13 to 0.54) relationships with GLA, TDW, and GY when all the experimental data were combined, but at the same time all SRIs failed to estimate the variation in WUE (**Tables 4 and 5**).

#### Multivariate statistical analysis to predict measured agronomic parameters

**Fig 5:**
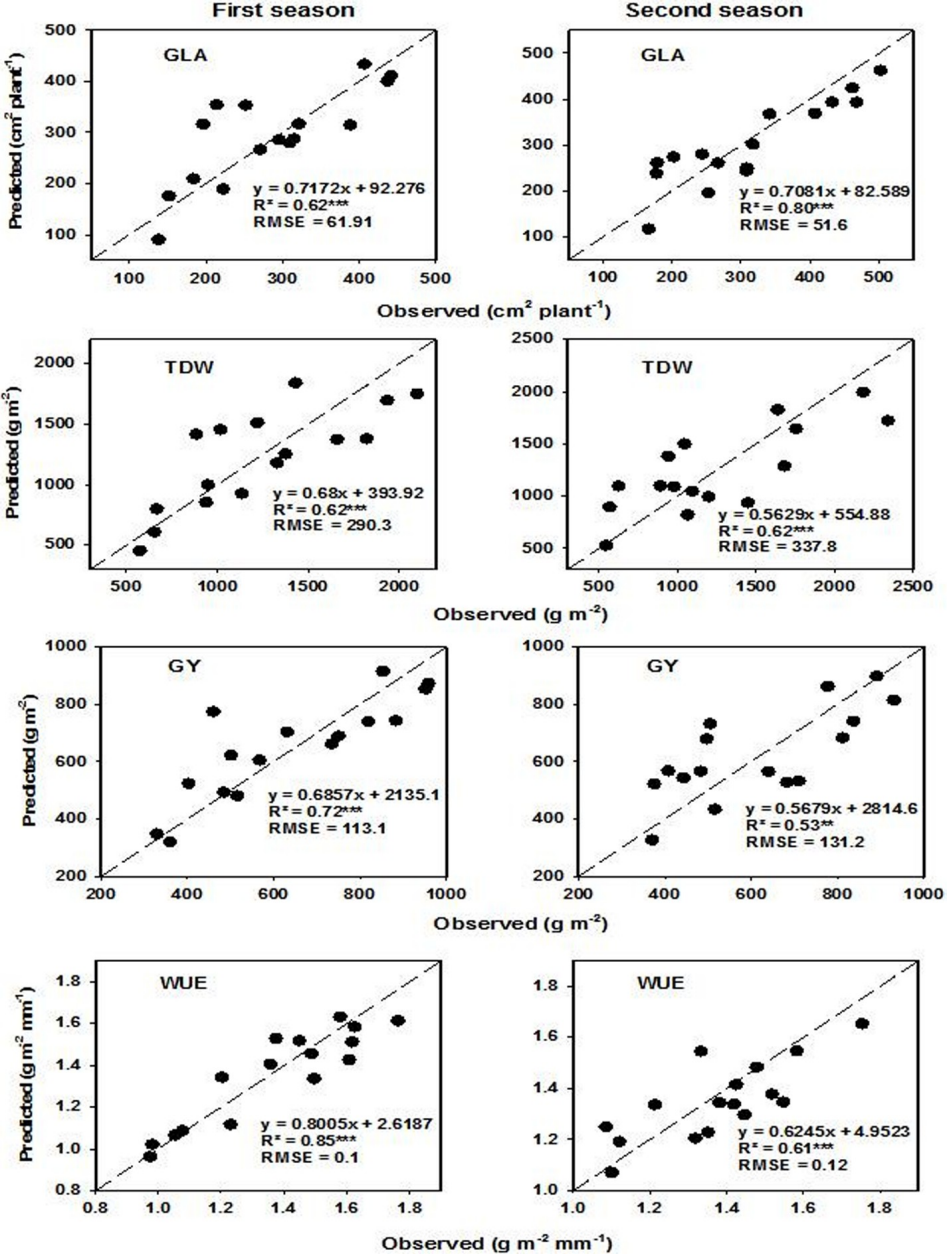
Scatter plots and linear relationships between observed and predicted values of green leaf area (GLA), aboveground total dry weight (TDW), grain yield (GY) and water use efficiency (WUE) based on PLSR for full spectrum regions (350–2500 nm) on a 1:1 line. The spectral reflectance data of the two seasons were used to predict the measured parameters during the first and second seasons.

**Fig 6:**
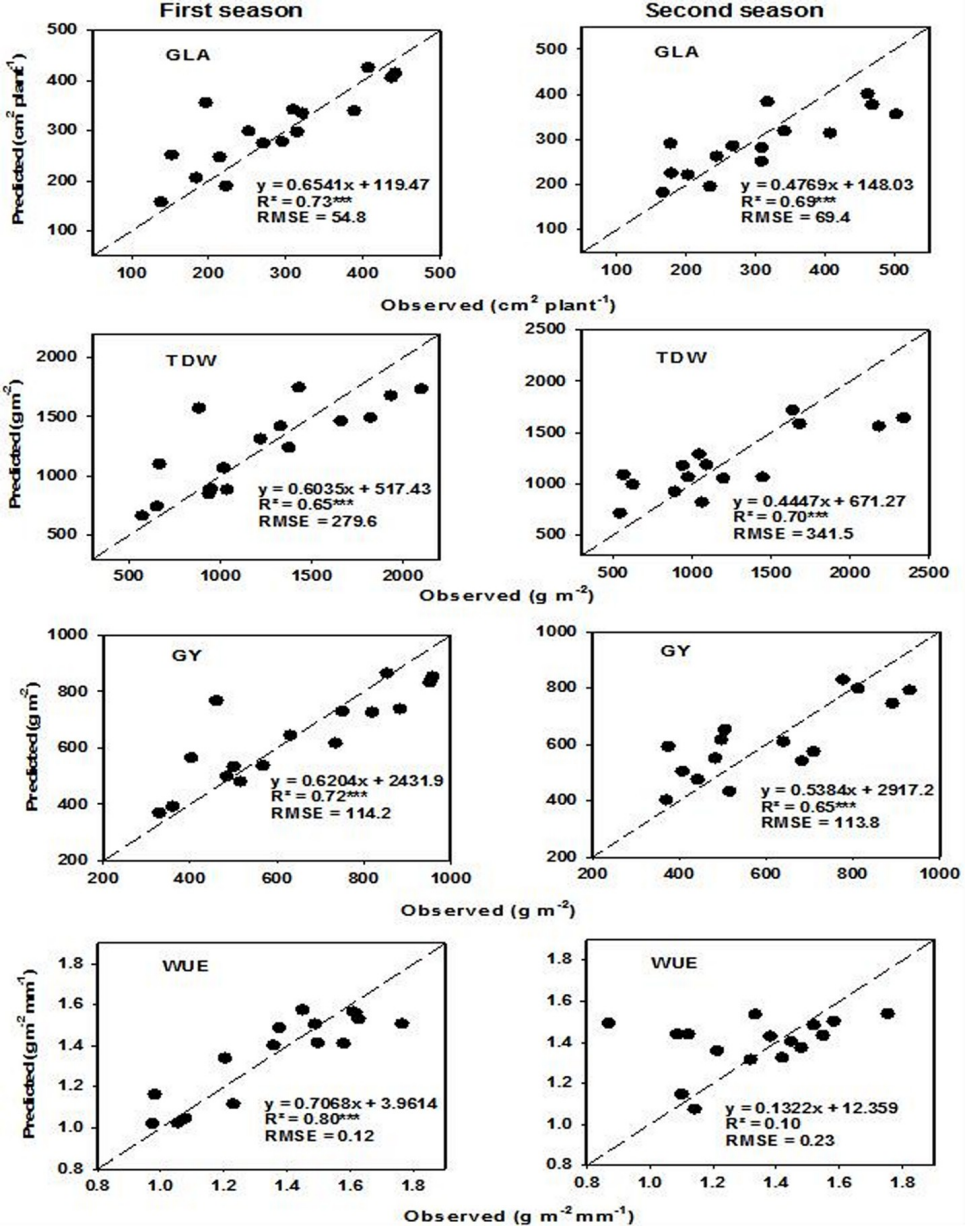
Scatter plots and linear relationships between observed and predicted values of green leaf area (GLA), aboveground total dry weight (TDW), grain yield (GY) and water use efficiency (WUE) based on SVM for full spectrum regions (350–2500 nm) on a 1:1 line. The spectral reflectance data of the two seasons were used to predict the measured parameters during the first and second seasons.

PLSR and SVM were applied as a cross-validation to select calibration models (**Table 6**) that were used to validate and predict measured parameters. The models were calibrated through cross-validation depending on the lower value of the RMSE. A suitable number of latent variables were selected using the dataset of all measured data and then validated using a dataset of samples from each irrigation rate and plant density (**Tables 7 and 8**), and for each season (**Figs 5 and 6**).

**Table 6:** Equations and the determination coefficients (R²) of partial least square regression (PLSR) and support vector machine (SVM) models that were used to predict different measured agronomic parameters (presented in Tables 7 and 8 and Figs 5 and 6).

Parameters	Equations	R^2^
**PLSR**		
**Green leaf area (GLA)**	y = 0.7034x + 90.195	0.70^***^
**Aboveground total dry weight (TDW)**	y = 0.6131x + 484.15	0.61^**^
**Grain yield (GY)**	y = 0.612x + 2467.3	0.61^**^
**Water use efficiency (WUE)**	y = 0.6131x + 5.2685	0.61^**^
**SVM**		
**Green leaf area (GLA)**	y = 1.1102x - 29.343	0.54^**^
**Aboveground total dry weight (TDW)**	y = 1.2077x - 239.73	0.58^**^
**Grain yield (GY)**	y = 1.1039x - 576.02	0.60^**^
**Water use efficiency (WUE)**	y = 1.0055x - 0.3783	0.45^*^

*, **, *** **indicate significance at 0.05, 0.01 and 0.001 P level, respectively**

**Table 7:** Predication models (the range for original and validation data of agronomic parameters (Par.), R², slope, intercept and RMSE) using partial least square regression (PLSR) for the full wavelength range (350–2500 nm). Models are based on the calibration data of two years for green leaf area (GLA), aboveground total dry weight (TDW), grain yield (GY) and water use efficiency (WUE) under individual irrigation rates and plant densities.

	Irrigation rates			Plant densities (D)				
Statistics	1.00 ET	0.75 ET	0.50 ET	D1	D2	D3	D4	D5
GLA (cm^2^ plant^-1^)								
Range of orginal data	315.3–510.4	252.5–377.0	138.9–244.6	178.2–407.9	221.7–461.8	197.0–486.8	152.7–510.4	138.9–432.8
Range of validation	287.2–461.8	243.1–367.3	90.0–393.2	209.1–368.5	249.8–433.2	274.0–461.8	175.3–410.9	90.0–393.2
**R**^**2**^	0.89^***^	0.46^*^	0.82^***^	0.87^***^	0.79^**^	0.83^***^	0.84^***^	0.75^**^
**Slope**	0.95	1.05	3.3	0.68	0.90	0.53	0.64	0.93
**Intercept**	21.6	-51.50	-364.6	96.0	21.7	171.0	97.6	- 0.25
**RMSE**	48.1	33.2	78.1	34.6	39.6	61.3	55.5	60.8
TDW (g m^-2^)								
Range of orginal data	950.3–2343.3	939.6–1683.4	545.9–1019.4	568.2–1047.2	978.3–1640.9	886.2–2186.0	626.9–2343.3	545.9–1823.6
Range of validation	997.1–1992.7	852.8–1509.8	450.4–1453.3	606.2–1497.4	934.5–1837.6	1093.7–1992.7	798.4–1749.0	450.4–1640.9
**R**^**2**^	0.59^*^	0.27	0.74^**^	0.66^*^	0.70^**^	0.74^**^	0.91^***^	0.88^***^
**Slope**	0.50	0.44	1.69	1.46	1.09	0.54	0.52	0.87
**Intercept**	750.8	616.8	-322.8	-186.6	45.2	654.4	531.4	61.4
**RMSE**	350.6	295.4	296.4	290.9	338.6	331.1	367.3	228.6
GY (g m^-2^)								
Range of orginal data	506.3–959.8	479.1–820.4	329.8–501.9	360.7–568.5	501.6–854.4	443.9–952.8	404.0–959.8	329.8–884.3
Range of validation	604.7–913.8	493.2–756.5	319.8–573.4	319.8–684.5	505.3–913.8	477.6–863.1	523.3–872.1	343.4–742.2
**R**^**2**^	0.60^**^	0.56^*^	0.68^**^	0.74^**^	0.83^**^	0.98^***^	0.80^**^	0.84^**^
**Slope**	0.53	0.63	2.08	1.42	0.92	0.77	0.60	0.70
**Intercept**	358.4	200.7	-357.9	-134.8	106.2	136.6	253.9	128.0
**RMSE**	114.69	103.46	128.97	100.22	80.64	141.0	114.64	109.21
WUE (g m^-2^ mm^-1^)								
Range of orginal data	0.86–1.60	1.05–1.79	1.01–1.53	0.86–1.16	1.31–1.63	1.35–1.79	1.23–1.61	1.01–1.48
Range of validation	0.96–1.55	1.06–1.65	1.02–1.53	0.96–1.41	1.30–1.58	1.20–1.65	1.33–1.63	1.02–1.46
**R**^**2**^	0.66^**^	0.83^***^	0.55^*^	0.74^**^	0.16	0.73^**^	0.88^***^	0.96^***^
**Slope**	0.74	0.71	0.68	2.27	0.45	0.80	0.61	0.87
**Intercept**	0.33	0.40	0.40	-1.28	0.76	0.21	0.60	0.15
**RMSE**	0.205	0.105	0.113	0.234	0.154	0.144	0.09	0.049

*, **, *** **indicate significance at 0.05, 0.01 and 0.001 P level, respectively. D1, D2, D3, D4, and D5 indicate plant density of 150, 250, 350, 450, and 550 seeds m**^**-2**^**, respectively.**

**Table 8:** Predication models (the range for original and validation data of agronomic parameters (Par.), R², slope, intercept and RMSE) using support vector machine regression (SVM) for the full wavelength range (350–2500 nm). Models are based on the calibration data of two years for the green leaf area (GLA), aboveground total dry weight (TDW), grain yield (GY) and water use efficiency (WUE) under individual irrigation rates and plant densities.

	Irrigation rates			Plant densities (D)				
Statistics	1.00 ET	0.75 ET	0.50 ET	D1	D2	D3	D4	D5
GLA (cm^2^ plant^-1^)								
Range of orginal data	315.3–510.4	252.5–377.0	138.9–244.6	178.2–407.9	221.7–461.8	197.0–486.8	152.7–510.4	138.9–432.8
Range of validation	233.0–425.4	251.1–383.7	157.1–355.4	205.5–318.8	246.3–425.4	221.6–405.6	225.3–414.7	157.1–339.43
**R**^**2**^	0.28	0.18	0.19	0.86^***^	0.86^***^	0.53^*^	0.81^**^	0.89^**^
**Slope**	0.45	0.60	0.81	0.51	0.74	0.45	0.53	0.38
**Intercept**	178.35	123.75	89.54	125.5	87.82	180.1	135.18	145.00
**RMSE**	93.4	36.04	72.27	45.1	34.49	73.9	64.1	33.2
TDW (g m^-2^)								
Range of orginal data	950.3–2343.3	939.6–1683.4	545.9–1019.4	568.2–1047.2	978.3–1640.9	886.2–2186.0	626.9–2343.3	545.9–1823.6
Range of validation	891.1–1745.3	849.6–1584.4	665.2–1571.4	745.2–1287.1	1063.4–1745.3	924.2–1676.1	993.6–1733.8	665.2–1491.5
**R**^**2**^	0.48^*^	0.71^**^	0.26	0.55^*^	0.91^***^	0.83^***^	0.87^***^	0.93^***^
**Slope**	0.39	0.69	0.73	1.16	1.13	0.56	0.42	0.67
**Intercept**	848.51	375.9	454.4	- 60.92	-42.86	505.20	718.60	365.7
**RMSE**	389.2	140.2	334.1	259.1	155.9	319.2	406.0	197.7
GY (g m^-2^)								
Range of orginal data	506.3–959.8	479.1–820.4	329.8–501.9	360.7–568.5	501.6–854.4	443.9–952.8	404.0–959.8	329.8–884.3
Range of validation	499.1–864.2	499.1–797.9	369.0–767.7	392.3–652.1	533.2–864.2	475.5–833.2	504.1–852.2	369.0–738.1
**R**^**2**^	0.63^**^	0.64^**^	0.34	0.48^*^	0.77^**^	0.92^***^	0.78^**^	0.96^***^
**Slope**	0.51	0.76	1.21	0.93	0.85	0.68	0.52	0.69
**Intercept**	343.34	111.5	139.6	89.3	104.0	182.0	286.9	156.6
**RMSE**	151.0	84.6	136.1	86.6	67.7	152.7	129.5	70.7
WUE (g m^-2^ mm^-1^)								
Range of orginal data	0.86–1.60	1.05–1.79	1.01–1.53	0.86–1.16	1.31–1.63	1.35–1.79	1.23–1.61	1.01–1.48
Range of validation	1.02–1.54	1.02–1.54	1.05–1.49	1.02–1.49	1.40–1.57	1.31–1.57	1.34–1.56	1.15–1.51
**R**^**2**^	0.75^**^	0.81^***^	0.70^**^	0.49^*^	0.02	0.52^*^	0.64^*^	0.84^***^
**Slope**	0.78	0.63	0.70	2.86	- 0.11	0.34	0.37	0.67
**Intercept**	0.33	0.46	0.43	-1.85	1.64	0.96	0.89	0.46
**RMSE**	0.095	0.14	0.01	0.21	0.12	0.15	0.12	0.089

*, **, *** **indicate significance at 0.05, 0.01 and 0.001 P level, respectively. D1, D2, D3, D4, and D5 indicate plant density of 150, 250, 350, 450, and 550 seeds m**^**-2**^**, respectively.**

The results showed that the PLSR models performed well for estimating all measured parameters under specific irrigation rate and plant density, with the exception of TDW under 0.75 ET, and WUE under D_2_. The R^2^ values for significant PLSR models ranged from 0.46 to 0.89, 0.66 to 0.98, and 0.53 to 0.85 under specific irrigation rate, plant density, and season, respectively (**Table 7 and Fig 5**). The SVM models also provided an accurate estimation of all measured parameters under specific irrigation rate and plant density, with the exception of GLA under the three specific irrigation rates, TDW and GY under 0.50 ET, and WUE under D_2_ and the second season. The R^2^ values for significant SVM models ranged from 0.48 to 0.81, 0.48 to 0.96, and 0.65 to 0.80 under specific irrigation rate, plant density, and season, respectively (**Table 8 and Fig 6**). These significant relationships for both models (PLSR and SVM) also delivered the lowest values for RMSE and the highest values for the slope (**Tables 7 and 8 and Figs 5 and 6**). In addition, the quality of PLSR and SVM for estimating measured parameters depended on the levels of irrigation rate and plant density as well as the season. In general, the PLSR models provided a more accurate estimation of GLA, TDW, and GY under 1.00 and 0.50 ET than those under 0.75 ET. The opposite held true for WUE. Under specific plant density, the PLSR models exhibited comparable values of R^2^ between the five plant densities, with the exception of TDW under D_1_ and WUE under D_2_, which showed moderate and non-significant relationships, respectively (**Table 7**)**.** The SVM models provided comparable values of R^2^ between 1.00 and 0.75 ET for GY and between the three irrigation rates for WUE but provided a more accurate estimation of TDW under 0.75 ET than under 1.00 and 0.50 ET. The SVM models exhibited comparable values of R^2^ between the five plant densities, with the exception of GLA under D_3_, TDW, GY and WUE under D_1_, as well as WUE under D_2_ and D_3_, which all showed moderately significant relationships (**Table 8**). Both models provided a more accurate estimate of measured parameters for the first season than for the second season, with the exception of GLA in the PLSR model and DW in the SVM model, which displayed inverse relationships (**Figs 5 and 6**).

## Discussion

The results of this study indicated that selecting the best combinations of irrigation rate and plant density could improve GY and water productivity of wheat under limited water supplies. These combinations could play an important role in regulating the amount of water lost by ET and the amount of water available to the plant by exerting positive or negative impacts on ground coverage at different stages of crop growth [**8,9,12**]. Combining a low irrigation rate with a very low plant density may provide sufficient water for individual plants, but results in more soil evaporation (E) due to less ground coverage, especially during early growth stages. To reduce ET, it was suggested to increase plant density under low irrigation rates to arrive at higher ground coverage, and this also might lessen the plant transpiration rate (*T*) because the plants may become shorter and have a fewer number of leaves under limited water supplies [**13**]. Although reducing plant density under an adequate irrigation rate might maximize dry matter production per plant, it does not add benefit for GY and decreases the WUE. All of these explanations indicate that the yield and water productivity of wheat could be improved by optimizing the best combinations of irrigation rate and plant density. The results of this study indicated that GLA, TDW, GY, and WUE responded strongly to different combinations of irrigation rate and plant density (**Fig 2**). Interestingly, a 25% reduction in the amount of water (0.75% ET) with medium plant density (D_3_) displayed a higher WUE than that obtained from combinations using full irrigation rate (1.00 ET) regardless of plant density, and produced a GY and sometimes a TDW that were similar to those obtained for 1.00 ET when combined with D_2_ or D_5_. A 50% reduction in the amount of water (0.50% ET) with D_2_ or D_3_ produced comparable values for most agronomic parameters with those obtained from 1.00 or 0.75 ET under low plant density (D_1_) (**Fig 2**).

The response of GY to different combinations of irrigation rate and plant density was tested through the slope of the regression (ky) between the relative GY decrease and the corresponding relative ET deficit. The ky values obtained for combined data (1.32 and 1.28 in the first and second seasons, respectively; **Fig 3**) in this study were higher than the value (1.15) reported for spring wheat by Doorenbos and Kassam [**45**]. Because ky values are strongly influenced by different crop management practices [**4,46**], the higher ky values found in this study may be due to the reduction in GY may not be offset under high plant density due to intense competition between plants and/or that the yield components of individual plants may not compensate for the decrease in plant density. Therefore, the values of ky could be somewhat improved under deficit water irrigation through the appropriate combinations of irrigation rate and plant density. Evidence for this hypothesis is evident from the wide range of ky values found among the different combinations of plant density and 0.75 and 0.50 ET treatments (**Table 3**). The results showed that a 25% (0.75 ET) and 50% (0.50 ET) water deficit may be acceptable for spring wheat productivity when the first treatment was combined with D_2_, D_3_ or D_4_, and the second treatment was combined with D_2_ or D_3_, as the ky values of these combinations were comparatively less than one or comparable with those obtained by Doorenbos and Kassam [**45**].

The linear regression analysis between GY and ET showed that about 71 and 64% of the variation in GY could be attributed to differences in seasonal ET in the first and second seasons, respectively (**Fig 4**). This also implies that the matching between irrigation rate and plant density has a significant influence on seasonal ET through manipulating ground coverage and adjusting canopy shading. A linear relationship between GY and seasonal ET has also been reported by Huang et al. [**47**] for winter wheat under different irrigation rates where about 66% of the GY variation is explained by seasonal ET.

Heretofore, and to the best of our knowledge, there are only a few studies that have examined the performance of hyperspectral reflectance sensing for estimating the variations in biophysical parameters under different combinations of multiple agronomic practices. As expected, manipulations between irrigation rate and plant density will create significant variation in different biophysical and biochemical characteristics of the canopy, which eventually induce significant changes in canopy reflectance characteristics. Feng et al. **[29]** reported that as the plant density of winter wheat increased, the spectral reflectance of the canopy in the VIS region decreased, while that of the NIR reflectance increased. The percentage of decrease in the VIS reflectance is predominantly linked to significant variations in leaf pigmentation and photosynthetic activity, while the percentage of increase in the NIR reflectance is associated with significant variation in biomass accumulation, canopy cover, green leaf area index (GLAI), leaf internal structure, and leaf water content [**41,42,48,49**].

Several studies have also reported that the wavelengths in the red-edge and NIR regions are considered to contain more information regarding GLAI and biomass than any other part of the spectral regions [**31, 50–52**]. Duan et al. [**53**] reported that under water-stressed conditions, the reflectance of red bands increased, while NIR reflectance decreased, and thus SRIs could provide important information about green biomass under stress. Because the leaf water content could be significantly affected by the combination of low or moderate irrigation rates with high plant density, the strong and weak water absorption bands found in the SWIR and NIR regions, respectively, cannot be ruled out. Therefore, the published and developed SRIs proposed for estimating measured agronomic parameters in this study included the three different spectral regions (VIS, NIR, and SWIR).

The majority of the SRIs examined in this study performed poorly in estimating the measured parameters under specific irrigation rates, but the performance improved significantly (R^2^ was between 0.47 and 0.99), with the exception of WUE, when all data of the irrigation rates were combined. Although all SRIs, with a few exceptions, showed low to moderate relationships with GLA, TDW, and GY (R^2^ was between 0.13 and 0.54), these SRIs were able to assess the variations in these parameters successfully when all data of the irrigation rates, plant densities, and seasons were combined (Tables 4 and 5). Very few SRIs exhibited a better fit with the measured parameters (R^2^ = 0.40–0.87) when all data of the plant density were combined. However, about half or more than half of the SRIs exhibited a better fit with GLA, TDW, and GY under D_1_ (R^2^ = 0.51–0.85), D_4_ (R^2^ = 0.50–0.99), and D_5_ (R^2^ = 0.50–0.84) when analyzing the relationship for each plant density separately (**Tables 4 and 5**).

All of these results indicate that different combinations of irrigation rate and plant density can create a large variation in the heterogeneity of canopy structure and architecture, LAI, soil background reflectance, light saturation, leaf angle distribution, and canopy chlorophyll and water content between treatments [**29,30,31,41,42,48,49**]. These comprehensive factors are sufficient enough to blur the relationships between most SRIs examined and the measured parameters. Therefore, several studies have reported that to improve the fit of the relationship between SRIs and measured parameters, it is important to analyze these relationships using the pooled data of all treatments in order to avoid the heterogeneity occurring between these treatments, or design new SRIs to remove the adverse effects of multiple factors on the spectral properties of the canopy [**29, 50, 54–59**]. For instance, Prabhakara et al. [**58**] reported that many SRIs failed to differentiate between the amount of biomass in six winter cover crops when the biomass was too high or too low. Once saturation or soil background reflectance was removed, the fit between SRIs and biomass was further improved when the data were analyzed across all levels of groundcover of the six crops. The index OSAVI, which was designed to reduce the influence of soil background, has been found to be effective for estimating biomass, chlorophyll content, and GLAI, although there is a wide range of variation between treatments in biomass and groundcover [**54,60–62**]. Lobos et al. [**57**] also reported that the relationships between SRIs and agronomic parameters showed a marked increase in the predictive potential when the data of all irrigation treatments were combined.

Although SRIs are easy to calculate and many of them were effective in estimating agronomic parameters, they are limited by their use of a few wavelengths, and are influenced by different degrees of soil background or saturation of the vegetation, as well as by timeliness and regional specificity [**33,63, 64**]. Previous studies have shown that multivariate regression models such as PLSR and SVM seem to be good alternatives to SRIs for interpreting the relationships between measured parameters and canopy spectral reflectance, which generally perform equally or better than SRIs for estimating the variations in these parameters [**23,26,33,34,63,65**]. The results of this study showed that the PLSR and SVM models performed better than the individual SRIs when estimating all measured parameters under specific irrigation rate and plant density (**Tables 7 and 8**). Similarly, Hansen and Schjoerring [**32**] obtained a better estimate of green biomass and leaf nitrogen concentration of winter wheat in a field experiment that included four nitrogen levels, two cultivars, and three different plant densities, when using PLSR rather than the best of the selected narrow-band SRIs. Zhai et al. [**66**] showed better performance for the SVM when estimating potassium, nitrogen, and phosphorus content in the leaves of different plants. The findings of these two studies and our results indicate that the PLSR and SVM may be useful tools for estimating the measured parameters when applied on hyperspectral reflectance data for different combinations of multiple agronomic practices which showing a great variation and heterogeneity in canopy reflectance characteristics.

## Conclusion

The traditional evaluation of growth, GY, and WUE by analysis of variance, ky, and the relationship between GY and seasonal ET demonstrated that the GY and water productivity of spring wheat could be improved by selecting the best combinations of irrigation rate and plant density under limited water supplies. The evaluation using hyperspectral data in this study indicated that this tool could be used as a rapid, cost-efficient, and non-destructive method for monitoring the growth, GY, and WUE of wheat under multiple agronomic treatments. Most of the SRIs assessed the measured parameters, excepting WUE, more efficiently when the data of three irrigation rates and the data of all experimental treatments were combined. A sufficient number of SRIs produced a satisfactory performance at the seedling densities D_1_, D_4_, and D_5_. The accuracy of hyperspectral data in estimating measured parameters improved when using multivariate analysis (PLSR and SVM), with the estimations provided by both models were better than those offered by individual SRIs under specific irrigation rates and some specific plant density.

## Supporting information

S1 Table Canopy spectral reflectance of irrigation rate and plant density in the range between 350 and 2500 nm of the spectrum. (XLSX)
