# Correction: Combining biophysical parameters, spectral indices and multivariate hyperspectral models for estimating yield and water productivity of spring wheat across different agronomic practices

**DOI:** 10.1371/journal.pone.0225294

**Published:** 2019-11-11

**Authors:** Salah El-Hendawy, Nasser Al-Suhaibani, Salah Elsayed, Yahya Refay, Majed Alotaibi, Yaser Hassan Dewir, Wael Hassan, Urs Schmidhalter

There is an error in affiliation 3 for author Salah Elsayed. The correct affiliation 3 is: Evaluation of Natural Resources Department, Environmental Studies and Research Institute, University of Sadat City, Menoufia, Egypt.
